# Seasonal Dynamics in Carbon Cycling of Marine Bacterioplankton Are Lifestyle Dependent

**DOI:** 10.3389/fmicb.2022.834675

**Published:** 2022-07-05

**Authors:** Sandra Martínez-García, Carina Bunse, Benjamin Pontiller, Federico Baltar, Stina Israelsson, Emil Fridolfsson, Markus V. Lindh, Daniel Lundin, Catherine Legrand, Jarone Pinhassi

**Affiliations:** ^1^Centre for Ecology and Evolution in Microbial Model Systems - EEMiS, Linnaeus University, Kalmar, Sweden; ^2^Departamento de Ecoloxía e Bioloxía Animal, Universidade de Vigo, Pontevedra, Spain; ^3^Institute for the Chemistry and Biology of the Marine Environment, University of Oldenburg, Oldenburg, Germany; ^4^Department of Functional and Evolutionary Ecology, University of Vienna, Vienna, Austria

**Keywords:** marine bacterioplankton, lifestyle, temporal dynamics, function, Baltic Sea

## Abstract

Although free-living (FL) and particle-attached (PA) bacteria are recognized as ecologically distinct compartments of marine microbial food-webs, few, if any, studies have determined their dynamics in abundance, function (production, respiration and substrate utilization) and taxonomy over a yearly cycle. In the Baltic Sea, abundance and production of PA bacteria (defined as the size-fraction >3.0 μm) peaked over 3 months in summer (6 months for FL bacteria), largely coinciding with blooms of *Chitinophagales* (*Bacteroidetes*). Pronounced changes in the growth efficiency (range 0.05–0.27) of FL bacteria (defined as the size-fraction <3.0 μm) indicated the magnitude of seasonal variability of ecological settings bacteria experience. Accordingly, 16S rRNA gene analyses of bacterial community composition uncovered distinct correlations between taxa, environmental variables and metabolisms, including *Firmicutes* associated with elevated hydrolytic enzyme activity in winter and *Verrucomicrobia* with utilization of algal-derived substrates during summer. Further, our results suggested a substrate-controlled succession in the PA fraction, from *Bacteroidetes* using polymers to *Actinobacteria* and *Betaproteobacteria* using monomers across the spring to autumn phytoplankton bloom transition. Collectively, our findings emphasize pronounced seasonal changes in both the composition of the bacterial community in the PA and FL size-fractions and their contribution to organic matter utilization and carbon cycling. This is important for interpreting microbial ecosystem function-responses to natural and human-induced environmental changes.

## Introduction

It is recognized that marine bacteria distinguished as free-living (FL, often defined as the size-fraction 0.2–3.0 μm) or particle-attached (PA, often defined as the size-fraction > 3.0 μm) diverge remarkably in terms of genomic content, phylogeny and physiology (e.g., DeLong et al., 1993; Lapoussière et al., 2011; Rösel and Grossart, 2012; Satinsky et al., 2014). Bacteria in these size-fractions differentially contribute to bulk community characteristics like abundance, production, and respiration, and therefore differentially influence element cycles in the ocean (e.g., carbon and oxygen; Smith et al., 1995; Riemann et al., 2000; Teira et al., 2010; Martínez-García and Karl, 2015). Understanding the contribution of FL and PA bacteria to the remineralization of dissolved and particulate organic matter (DOM and POM, respectively) is important in marine ecology in general, as well as in the context of global change, also because particles formed in surface waters comprise a major portion of the sinking organic material that constitutes the biological carbon pump, transporting carbon from the surface to deep waters (Ducklow et al., 2001; Grossart et al., 2003). Microbes with different life strategies may therefore contribute differently to biological transformation of DOM and POM (Grossart, 2010); and while FL bacteria are typically more abundant than PA bacteria in the water column, the latter can still contribute prominently to productivity and enzymatic activity measurements because of higher per cell metabolic rates (Smith et al., 1995; Riemann et al., 2000). Variability in biotic and abiotic factors is thought to control not only the abundance and activity of PA and FL bacteria, but also the ratio between them over short time scales (Mével et al., 2008).

Several studies have shown that the taxonomic composition of FL and PA fractions of bacterial communities differs, with FL bacteria often being dominated by for example *Alphaproteobacteria* (e.g., SAR11 and the *Roseobacter* clade) while certain lineages of *Gammaproteobacteria*, *Planctomycetes* and *Bacteroidetes* are predominantly found in the PA fraction (DeLong et al., 1993; Acinas et al., 1997; Ghiglione et al., 2007; Milici et al., 2016 among others). Furthermore, particles of different sizes host bacterial communities with different taxonomic composition (Kellogg and Deming, 2014; Mestre et al., 2017) and there is also evidence of stochasticity in particle colonization (Bižić-Ionescu et al., 2018). Nevertheless, there are reports on similar taxonomic distributions among FL and PA bacteria (e.g., Noble et al., 1997; Hollibaugh et al., 2000), potentially as a consequence of the transfer of bacteria from one size-fraction to the other (Bižić-Ionescu et al., 2015), suggesting that the composition in the two size-fractions may differ between environments or may change over time.

It is hypothesized that the activity of PA bacteria on particles and in microaggregates results in the release of labile substrates with high nutritional value to the FL bacterial community (Herndl, 1988; Chrost, 1991; Azam et al., 1994). Thereby, dissolved organic matter utilization by the bacterial community is in part controlled by an interplay between PA and FL bacteria, where differences in the activity of extracellular enzymes and the utilization of different substrates between the two compartments vary in relation to environmental conditions, substrate availability, and microbial community structure (e.g., Sala et al., 2005; Arnosti et al., 2012; Ruiz-González et al., 2015; Baltar et al., 2016).

Nevertheless, studies differ substantially regarding the similarity or divergence of hydrolytic enzyme activity and substrate utilization of PA and FL bacterial communities, such as temporal patterns in substrate spectrum or specific activities (Becquevort et al., 1998; Bickel and Tang, 2014; D'ambrosio et al., 2014; Zhu et al., 2016). This may be linked to differences in the specific environmental settings of different locations and between laboratory experiments. Overall, these considerations suggest that field studies investigating temporal and/or spatial variability of PA and FL ectoenzymatic activity and substrate utilization may shed light on the roles of PA and FL bacteria in the transformation of DOM.

The effects of environmental factors on bulk bacterioplankton characteristics have been widely investigated (e.g., Alonso-Sáez et al., 2007; Lomas et al., 2013; Martínez-García and Karl, 2015; Bunse et al., 2019). However, while many studies have focused on specific aspects of experimental communities or responses on short temporal scales (e.g., Becquevort et al., 1998; Riemann et al., 2000; Lapoussière et al., 2011; Rösel and Grossart, 2012), only a few have investigated the temporal variability in size-fractionated bacterial abundance or rates on longer time scales, e.g., across seasonal cycles (e.g., Becquevort et al., 1998; Riemann et al., 2000; Lapoussière et al., 2011; Rösel and Grossart, 2012).

Therefore, the aim of the current study was to determine the variability in activity and community composition of PA and FL bacteria during a complete annual cycle sampled roughly fortnightly at the Linnaeus Microbial Observatory (LMO), Baltic Sea, using V3V4 16S rRNA gene amplicons. We reasoned that studying the coupling of distinct bacterial compartments with changes in physicochemical conditions and phytoplankton variability would help understanding microbial functioning, particularly in temperate waters subjected to pronounced seasonality (Pinhassi et al., 2006; Teira et al., 2015; Bunse et al., 2019).

## Materials and Methods

### Field Sampling and Location

Unfiltered natural surface seawater was collected nearly twice a month starting from March 2015 until April 2016 at the Linnaeus Microbial Observatory located 10 km off the east coast of the Island Öland in the Baltic Sea Proper (N56°55.851, E17°03.640), at 2 m depth (maximum depth 40 m, with important thermal stratification from May/June to October (Legrand et al. (2015))) at *ca.* 9 a.m., using a Ruttner sampler (Legrand et al., 2015). Water was collected in acid washed, MilliQ-rinsed polycarbonate bottles and transported back to the laboratory within 1 h. In the laboratory an aliquot of the sample was gently filtered (gravity filtration) through a 3 μm pore size polycarbonate filter (Poretics). The filtrate is considered here as the free-living (FL) fraction and the particle-attached (PA) fraction corresponds to bacteria retained in the 3 μm pore size filter. Seasons in the Baltic Proper were defined according to HELCOM as follows; Spring: March–May, Summer: June–September, Autumn: October–December, and Winter: January–February (Wasmund et al., 2011).

### Temperature, Salinity, and Nutrients

Temperature and salinity were measured on site. In addition, temperature, salinity, and light intensity throughout the water column were measured using a CTD probe (AAQ 1186-H, Alec Electronics, Japan). Samples for total inorganic nutrients (NO_3_^−^ and NO_2_^−^ (together presented as nitrate, NO_3_^−^), NH_4_^+^, PO_4_^−3^ and SiO_3_) were collected and frozen until analysis using colorimetric methods (UV-1600 Spectrometer, VWR; Valderrama, 1995). Samples for dissolved organic carbon (DOC) were collected in duplicates; 20 ml samples were filtered through precombusted (450°C, 2 h) GF/C glass fiber filters (pore size ~1.2 μm) *via* gravity, acidified (200 μl HCl (2 M)) and stored in precombusted glass vials (475°C, 3 h) with acid washed lids at 6–8°C until analysis. DOC concentration was measured *via* high temperature catalytic oxidation (HTCO) using a Shimadzu TOC-V analyzer and a Shimadzu TOC-L Total Organic Carbon Analyzer following Traving et al. (2017). A dilution series of acetanilide was used as a standard, in accordance with Cauwet (1999) and the detection limit for DOC measurements was at or below 10.8 μM.

### Chlorophyll *a* and Bacterial Abundance

Chlorophyll *a (*Chl *a*) concentrations were measured from duplicates of 500 ml seawater filtered on A/E glass fiber filters (Pall Laboratory) extracted in 96% EtOH overnight and analyzed using a Trilogy fluorometer (Turner Designs, United States) following the protocol from Jespersen and Christoffersen (1987). Seawater samples for bacterial abundances (BAs) were preserved with formaldehyde (2% final concentration) as described in Lindh et al. (2015) and enumerated with a Cube8 flow cytometer (Partec, Germany) as described in Del Giorgio et al. (1996). Samples for heterotrophic bacteria determination were stained using Sybr Green DNA fluorochrome and heterotrophic bacteria were distinguished based on their green fluorescence (FL1, 530 nm) and side scatter. Cell concentrations were measured in the <3 μm filtrate (here considered as the free-living fraction), in raw seawater and also in raw seawater subjected to 10 s of sonication (Bandelin Sonorex). Sonication procedure was adapted from (Lam and Cowen, 2004) and validated by microscopy direct counts (Supplementary Figure S1). The optimum sonication time (that which promoted the maximum number of intact cells) was set up after different experiments with natural microbial communities and cultures (see Supplementary Figure S1). Cell concentration in the PA fraction was calculated as the difference between the value in the raw water subjected to sonication and the value in the raw water. Sonication time was optimized to maximize the number of detachable and intact cells (Supplementary Figure S1).

### Bacterial Heterotrophic Production (BP)

The ^3^H-Leucine incorporation method (Kirchman et al., 1985) modified as described by Smith and Azam (1992), was used to determine leucine (leu) incorporation rates after 2 h incubation. The final concentration of leucine used was 40 nM. A conversion factor of 0.86 for the transformation from cellular carbon to protein (Simon and Azam, 1989), a factor of 0.073% leucine in total proteins (Simon and Azam, 1989) and a theoretical conversion factor for internal isotope dilution of 2 were used according to Simon and Azam (1989). Average values of technical replicates (*n* = 3) are presented. BP in the PA fraction was calculated as the difference between BP in the raw sample and BP in the 3 μm filtrate.

### Microbial Respiration (R)

*In vivo* electron transport system (ETS) activity rates were used as an estimator of microbial respiration following Martínez-García et al. (2009). Five 250 ml dark bottles were incubated at *ca. in situ* temperature during 4 h. Initial time-course experiments (incubation times up to 20 h) were performed to determine the optimal incubation time and the single 4 h procedure was used throughout the study. After incubation, samples were filtered sequentially through 3 and 0.2 μm pore size polycarbonate filters (Poretics). FL bacterial respiration was operationally defined as ETS activity between 0.2 and 3 μm. To transform ETS activity into C respiration, a R/ETS ratio of 12.8 (Martínez-García et al., 2009) and a respiratory quotient (RQ) of 0.8 (Williams and Del Giorgio, 2005) were used. The contribution of FL bacterial communities to total respiration was calculated as %R-FL = [R-FL / (R-FL + R-PA)] × 100.

### Extracellular Enzymatic Activity

Extracellular enzymatic activity (EEA) of leucine aminopeptidase (LAPase), β-glucosidase (BGase) and alkaline phosphatase (APase) was determined in technical quadruplicates according to the fluorometric enzyme assays and conditions described in Baltar et al. (2016). The hydrolysis rates of fluorogenic substrate analogs L-leucine-7-amido-4-methylcoumarin (MCA), 4-methylumbelliferyl (MUF)-β-D-glucoside and MUF-phosphate were analyzed to estimate potential activity rates of LAPase, BGase, and APase, respectively (Hoppe, 1983). Final substrate concentrations, based on previous kinetic determinations from LMO waters (Baltar et al., 2016), were 32.25 for MUF-BGase, 100 uM for MUF-APase and 500uM for MCA-LAPase. Plates were incubated 1 h at *ca. in situ* temperature in dark conditions and were measured before and after the incubation. Subsamples for blanks were filtered through 0.2 μm pore size low protein binding filters (Acrodisc, Pall) and fluorescence values blank were corrected. Averages of technical quadruplicates are shown. EEA in the FL fraction was measured in the <3 μm filtrate and EEA in the PA fraction was calculated as the difference between the value in raw water and the value in the <3 μm filtrate. EEA in the FL fraction includes cell-free (dissolved) activity (Baltar et al., 2016).

### Carbon Compound Utilization

Plates (Biolog EcoplateTM, Biolog Inc.) were inoculated with 150 μl sample in each well and immediately read at a wavelength of 590 nm on a microwell plate reader (FLUOStar, BMG Labtech). Biolog plates were incubated at *ca. in situ* temperature for 2–3 weeks and read regularly (every 1–2 days) until maximum average well color development (AWCD) was reached (Garland et al., 2001). The utilization of each carbon source was expressed as their respective absorbance value normalized by AWCD (Sala et al., 2010). Averages of technical triplicates are shown. Carbon compound utilization in the FL fraction was measured in the <3 μm filtrate and carbon compound utilization in the PA fraction was calculated as the difference between the value in raw water and the value in the <3 μm filtrate.

### Prokaryotic Community Composition

For bacterial community composition estimates, 3-8 L seawater were pre-filtered through 3.0 μm pore size, 47 mm diameter, polycarbonate filters (Pall life sciences; 3 μm fraction, PA). Smaller and free-living (3–0.2 μm, FL fraction) bacterioplankton biomass was subsequently collected on 0.2 μm Sterivex™ cartridge filters (Millipore) and filters were stored frozen at −80°C in TE buffer. DNA was extracted from the biomass collected in the filters using the phenol-chloroform protocol (one extraction round) described by Boström et al. (2004) and modified after Bunse et al. (2016). DNA was extracted from the Sterivex filters (free-living fraction) and from the 3.0 μm pore size filters (particle-attached fraction). The V3V4 region of the 16S rRNA gene—well established for bacterial diversity studies in the Baltic Sea by our laboratory as well as others (e.g., Herlemann et al., 2011; Hugerth et al., 2014; Lindh et al., 2015)—was amplified in PCRs using the primer pair 341f-805r as described in Herlemann et al. (2011) and Hugerth et al. (2014). PCR amplicons were generated in duplicates. DNA concentration was analyzed using a Nanodrop or Qubit 2.0 Fluorometer (Life Technologies) and amplicon specificity was tested by gel electrophoresis. Sequencing was carried out at the Science for Life Laboratory (Sweden), on the Illumina MiSeq platform, producing 2 × 300bp paired-end reads.

### Denoising and Taxonomic Annotation of 16S rRNA Gene Amplicon Reads

Raw sequence reads were denoised and taxonomically annotated with nf-core Ampliseq (Ewels et al., 2020; Straub et al., 2020) v. 1.1.3, a Nextflow (Di Tommaso et al., 2017) workflow that uses cutadapt (v. 2.6, Martin, 2011) to identify reads with primers and remove them, QIIME2’s (v. 2019.10.0, Bolyen et al., 2019) DADA2 implementation (Callahan et al., 2016) to denoise amplicon reads and remove bimeras, using default parameters except that forward reads were trimmed to 259 bp after removal of primers, and reverse reads to 199 bp before denoising. Subsequently, QIIME2’s Bayesian classifier was used to assign taxonomy to the amplicon sequence variants (ASVs). The SILVA (Quast et al., 2012) database release 132 was used as the taxonomy reference database.

### Statistical Analyses and Graphical Outputs

R (version 4.1.0, RStudio Team, 2021) was used for statistical analyses and graphical outputs. To normalize for sequencing bias and compositionality of the data and for downstream statistical analyses, centered log ratio (clr) transformation was applied to bacterial community composition of raw counts with the codaseq.clr and aldex.clr functions after zero-count normalization (CoDaSeq and ALDEx2 packages, respectively; Fernandes et al., 2013; Gloor et al., 2016a,b). The influence of seasonality and filter fraction on bacterial community composition was tested using a permutational multivariate analysis of variance (PERMANOVA) with the function adonis (vegan package; Oksanen et al., 2020). Correlations between the change in bacterial community composition (Bray-Curtis distances) and the change in environmental variables was computed with Mantel tests with the mantel function (vegan package). A significance value of *p* < 0.05 was considered for both PERMANOVA and Mantel tests. The heatmap.2 function (gplots package) was used to obtain clusters (dist function, Euclidean method) within the 152 most abundant bacteria (selected as the top 100 ASVs in each fraction that have the highest abundance summed in all samples). Correlation analysis between bacteria and biotic and abiotic variables was performed with the aldex.corr function (ALDEx2 package) following data normalization suggested in package documentation. Spearman’s rank correlations were computed and a significance value of *p* < 0.05 was considered. Richness and diversity indexes were calculated after subsampling to the lowest number of reads present in a sample using the rarefy function (vegan package). Heatmaps were plotted with the Heatmap function (ComplexHeatmap package, Gu et al., 2016) and relative abundance bar-plots by means of the ggplot package (Wickham, 2016). Line plots were made with the Sigmaplot software (Systat Software, Inc.).

## Results and Discussion

### Physicochemical Factors and Chlorophyll *a* Concentrations

**Figure 1:**
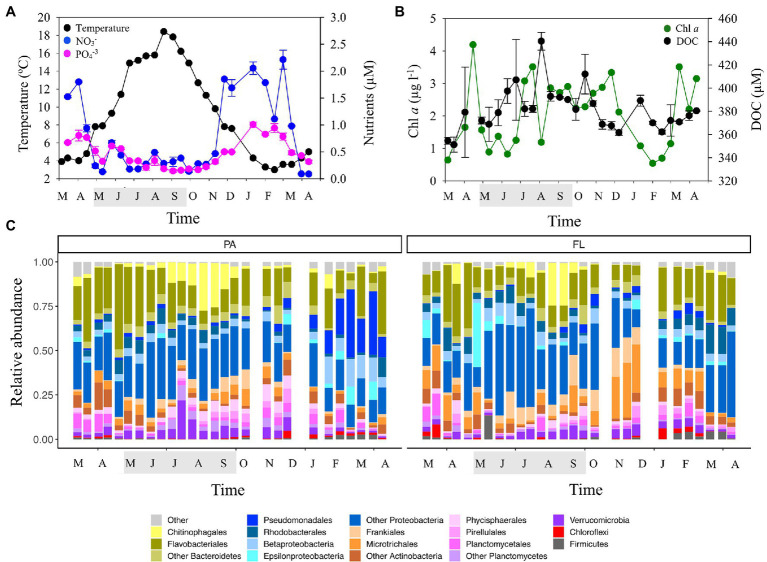
Temporal evolution of: **(A)** temperature, nitrate, and phosphate concentrations, **(B)** chlorophyll *a* (Chl *a*) and dissolved organic carbon (DOC) concentrations, and **(C)** taxonomic composition of bacterial communities in the two size-fractions studied: >3 μm (particle-attached; PA) and <3 μm (free-living; FL). The shaded period in the x-axis corresponds to summer.

The overall seasonal dynamics in environmental conditions registered during the study period agreed with previous reports from LMO (Legrand et al., 2015; Lindh et al., 2015; Bunse et al., 2019). Temperatures ranged from 3.0°C in winter to 18.4°C in summer (Figure 1A). Chlorophyll *a* (Chl *a*) concentration, as proxy for phytoplankton biomass, showed typical temporal dynamics with spring blooms up to ~4.2 μg l^−1^ and a summer bloom up to 3.5 μg l^−1^ in 2015 that extended into autumn (Figure 1B). Dissolved organic carbon (DOC) concentration also showed seasonal dynamics with higher values during summer and autumn up to 440 μMC (Figure 1B). Inorganic nutrient concentrations decreased during spring and into summer, through consumption during the phytoplankton blooms (Legrand et al., 2015), while higher concentrations were observed from November and into winter (Figure 1A; Supplementary Figure S2).

### Temporal Dynamics in Bacterioplankton Community Composition

Changes in the relative abundance of major bacterial taxonomic groups were observed throughout the year in both the FL and PA fractions of the bacterial community (Figure 1C). In the PA fraction, Flavobacteriales were dominant through spring (up to ~50%) being successively replaced by Chitinophagales in summer from July through September. Verrucomicrobia and Planctomycetes, which were relatively more abundant in the PA than in the FL fraction, peaked in summer and in autumn/winter, respectively. All these groups are known to be associated with diatom and cyanobacteria blooms in the Baltic Sea and elsewhere (Eiler and Bertilsson, 2004; Herlemann et al., 2013; Lindh et al., 2015; Bunse et al., 2016; Zhu et al., 2016). Actinobacteria (Frankiales and Microtrichales) were enriched in the FL fraction in spring and autumn, with lower relative abundances in summer. In contrast, Rhodobacterales increased during the summer period (Figure 1C), which is in accordance with previous studies reporting increases of this group during phytoplankton blooms (e.g., Taylor et al., 2014).

Bacterial community composition differed between size-fractions (PERMANOVA, *p* < 0.001) and sampling season was a significant factor driving compositional changes in both the PA and FL fractions (PERMANOVA, *p* < 0.001). Furthermore, the size-fraction effect on bacterial community composition differed by season (PERMANOVA, *p* < 0.001). Correlation analyses between distance matrices of environmental variables and bacterial community composition showed that changes in the composition of PA bacteria were significantly correlated with changes in temperature (Mantel test, *p* < 0.05; Supplementary Table S1). Bacterial diversity was generally higher in the PA than the FL fraction (Supplementary Figure S3), as observed also in other seas (Crespo et al., 2013). However, short-term dynamics in alpha diversity in both the PA and FL fractions were observed throughout the seasonal cycle, often coinciding with the relative increase in specific bacterial groups. As an example, an increase in the relative abundance of FL Epsilonproteobacteria in May 2015 coincided with a marked decrease in alpha diversity and also a sharp decrease in chlorophyll *a* concentration (Figures 1B,C; Supplementary Figure S3). These periods of low phytoplankton abundance have been previously described as “clear water-phase periods” (period of low phytoplankton biomass often resulting from grazing processes after phytoplankton blooms, Lampert et al., 1986). The temporal changes in alpha and beta diversity were accompanied by dynamics in microbial activity in this system (i.e., bulk productivity, and respiration, exoenzymatic activity and functional diversity), suggesting pronounced linkages to the carbon and nutrient budgets within the microbial compartment.

### Bacterial Abundance and Production

**Figure 2:**
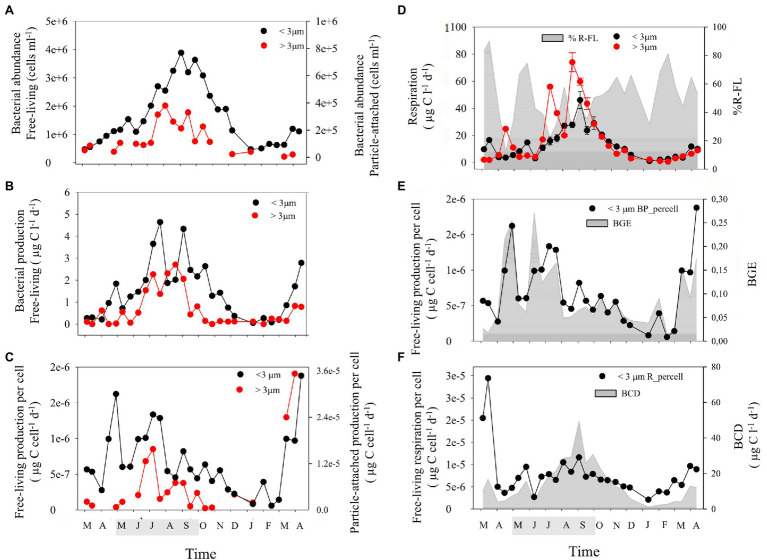
Temporal evolution of: **(A)** abundance of FL and PA bacteria, **(B)** heterotrophic bacterial production in FL and PA size-fractions, **(C)** production per cell in FL and PA bacteria, **(D)** respiration in FL and PA bacteria and contribution of FL bacteria to total respiration (%R-FL, gray shading), **(E)** bacterial production per cell and bacterial growth efficiency (BGE) in FL bacteria, and **(F)** bacterial respiration per cell and bacterial carbon demand (BCD) in FL bacteria. Note the different scales on additional y-axes in panels **A**,**C**,**D**,**E**, and **F**. Data for the FL and PA size-fractions are indicated by <3 μm and >3 μm, respectively. The shaded period in the x-axis corresponds to summer.

Bacterial abundance (BA) and Bacterial production (BP) in the two size-fractions displayed a seasonal cycle with numbers building up during spring, peaking in summer (up to 3.9 × 10^6^ and 3.8 × 10^5^ cells ml^−1^ in BA-FL and BA-PA, respectively, and up to 3.5 and 2.03 μg C l^−1^ d^−1^ in BP-FL and BP-PA, respectively) and decreasing again through autumn (Figure 2A). Both BA-FL and BA-PA were positively correlated with temperature and DOC concentrations (Supplementary Table S2) indicating a coupling between the two bacterial compartments and resource availability. However, the temporal dynamics in abundance and production differed substantially between fractions, with both BA and BP in the PA fraction displaying a shorter period of elevated levels (from mid-summer to mid-autumn) compared to BA and BP in the FL fraction (complete summer and autumn periods; Figures 2A,B). Curiously, the few published works comparing temporal dynamics in abundance and production of FL and PA bacteria also show differences in dynamics between the fractions, both in the field (Becquevort et al., 1998; Rösel and Grossart, 2012) and during induced phytoplankton blooms in the laboratory (Smith et al., 1995; Riemann et al., 2000; Worm et al., 2001; Grossart et al., 2006). Interestingly, in the present work, the period of elevated BA-PA and BP-PA coincided with an important increase in the relative abundance of Chitinophagales and with an increase in phytoplankton biomass (Figures 1B,C). This suggests that Chitinophagales may have an important role as degraders of complex organic compounds during the colonization and hydrolysis of particles from phytoplankton blooms in this ecosystem. By contrast, increased bacterial abundance and activity in the FL fraction extended before and after the phytoplankton biomass maximum. This suggests a sequential utilization of distinct pools of organic matter: FL heterotrophic bacteria (like Frankiales and Microtrichales, Figure 1C) would benefit from DOM released by both algal exudation from the beginning of the bloom and cell lysis after bloom breakdown. On the other hand, PA bacteria (like for example Chitinophagales and Verrucomicrobia, Figure 1C) would probably actively exploit phytoplankton cells upon algal colonization and therefore would benefit from shorter periods with elevated phytoplankton biomass, that is, once primary production allows cell accumulation and before bloom breakdown occurs.

Per cell activities in the PA fraction (BP-PA per cell) were on average one order of magnitude higher (from 0.5 to 36-fold) than in the FL fraction (BP-FL per cell). Interestingly, during the productive period the cell-specific production (which peaked in spring and early summer) increased before bulk production (which peaked in summer) in both size fractions (Figures 2B,C). This suggests an upregulation of cell-specific metabolism of bacterial communities in both size fractions as a response to changes in resource availability in the field. Although PA bacteria are generally recognized to display higher cell-specific rates than FL bacteria, few previous works demonstrate this by comparing cell-specific production rates of PA and FL bacteria during phytoplankton blooms in the field or in mesocosms (e.g., Smith et al., 1995; Becquevort et al., 1998; Riemann et al., 2000). To our knowledge, this is the first work determining the dynamics of cell-specific production rates of FL and PA bacteria across seasons over a full year. Our results suggest a particularly high contribution of PA compared to FL cells to carbon cycling in this system during summer.

Sharp decreases in bulk and per cell BP-FL were observed during May 2015 that coincided with a decrease in the bacterial diversity in the FL fraction and with the above-mentioned increase in Epsilonproteobacteria (Figures 1C, 2C; Supplementary Figure S3). Also during spring 2016, diversity was higher in the FL than in the PA fraction, coinciding with the substantial increases in BP-FL and BP-FL per cell during that period (Figures 1C, 2C; Supplementary Figure S3). These results confirm that temporal changes in size-fractionated alpha and beta diversity were accompanied by dynamics in both specific and bulk microbial activity in this system.

### Microbial Respiration

Microbial respiration (R) in both size-fractions increased from very low values in spring to peaks in summer/early autumn (highest values in the PA fraction; up to 74.0; μg C l^−1^ d^−1^) and then steadily decreased into winter (down to 0.6 μg C l^−1^ d^−1^; Figure 2D). An important control of temperature on microbial respiration was observed, particularly after the summer phytoplankton bloom (Figure 2D), that is likely resulting from the strong temperature effect on cellular metabolism (Pomeroy and Wiebe, 2001; Robinson, 2008). These observations confirm the role of environmental conditions, particularly temperature and resource availability, in controlling microbial respiration in both size-fractions in the Baltic Sea (see review in Robinson, 2008; Martínez-García and Karl, 2015; Teira et al., 2015).

The average contribution of FL bacterioplankton respiration to total community respiration was highly variable (%R-FL = 12%–90%, Figure 2D) and ranged from values reported for oligotrophic systems dominated by picoplankton to values in coastal waters in which larger microplankton are prevalent (Robinson and Williams, 2005; Robinson, 2008 and references therein). This indicates the importance of season and trophic status on FL bacterioplankton respiration rates (Sherry et al., 1999; Lemée et al., 2002). R-PA increased during the summer period suggesting a seasonal contribution of PA bacteria and larger planktonic organisms (including large-sized and chain-forming eukaryotic phytoplankton plus filamentous cyanobacterial taxa) to total community respiration during phytoplankton blooms. R-FL per cell reached higher values during spring and late summer (Figure 2F) and was significantly correlated with beta diversity of FL bacteria (Mantel test, *p* < 0.05; Supplementary Table S1), suggesting that changes in carbon cycling through the microbial compartment are intricately linked to community composition.

### Bacterial Growth Efficiency and Carbon Demand

Bacterial growth efficiency in the FL fraction (BGE-FL) was high (0.21–0.27) during and following the spring and summer phytoplankton blooms. In contrast, relatively low and stable BGE levels (below 0.09) were registered during autumn and winter (September–February; Figure 2E). During spring and early summer, important changes were registered in BP-FL per cell that did not coincide with changes in R-FL per cell, but both variables were tightly coupled from summer to winter, which is seen as highly stable BGE-FL (Figures 2E,F). In contrast, bacterial carbon demand in the FL fraction (BCD-FL) ranged from 1.1 to 49.3 μg C l^−1^ d^−1^ and was correlated (Spearman’s test, *p* < 0.05) with temperature following the seasonal pattern of R-FL and R-FL per cell (Figure 2F; Supplementary Table S2).

Interestingly, the range in bacterial growth efficiency values observed here over a year at a single location in the Baltic Sea (0.05–0.27, Figure 2E) is comparable to the range of values recorded for distinct marine environments differing greatly in productivity (Del Giorgio and Cole, 1998; Reinthaler and Herndl, 2005; Alonso-Sáez et al., 2007; Martínez-García et al., 2010a,b, 2018). It is interesting to note here that, to our knowledge, this is the first study in which bacterial growth efficiency is calculated from bacterial production and respiration measured at the same time from aliquots of the same sample filtered through a 3-μm pore size filter, as bulk production has been traditionally used. The size-fractionation of bacterial production shown here avoids overestimation of bacterial growth efficiency due to elevated algal cell accumulation. In our study, the BGE-FL quickly responded to changes in the rate of supply and the quality of the substrates in accordance with previous studies from different marine environments (Lemée et al., 2002; Martínez-García et al., 2018), in freshwater (Findlay et al., 1992; Schweitzer and Simon, 1995), and in the laboratory (Coffin et al., 1993). An example of this would be the sharp decrease in growth efficiency (down to 0.06) during the “clear water-phase period” observed in May 2015. The changing environmental conditions in the Baltic Sea throughout the year facilitate intense phytoplankton blooms in spring and early summer that are capable to promote very high bacterial growth efficiencies. On the other hand, the decrease in light availability and temperature causes low primary productivity which along with the scarcity of resources during autumn and winter leads to a decrease in bacterial growth efficiency. Our findings show that the different ecological settings that bacterioplankton are exposed to during a yearly cycle in a temperate sea induce pronounced adjustments in their metabolism, which can be detected through the display of a wide spectrum of cell-specific production rates and growth efficiencies.

### Extracellular Enzymatic Activity and Specific Carbon Source Utilization

**Figure 3:**
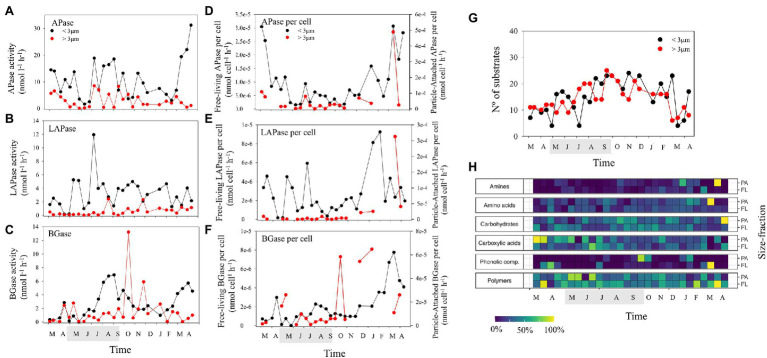
Temporal dynamics in bulk and per cell extracellular enzymatic activities of: **(A,D)** alkaline phosphatase (APase), **(B,E)** leucine aminopeptidase (LAPase), and **(C,F)** β-glucosidase (BGase). Temporal dynamics in **(G)** the number of substrates used, and **(H)** the relative substrate use in the two size-fractions studied (normalized per substrate). The shaded period in the x-axis corresponds to summer.

EEAs in the FL fraction were generally higher than in the PA fraction, although per cell values were on average one order of magnitude higher for the PA fraction (Figures 3A–F). The seasonal dynamics of alkaline phosphatase (APase), leucine aminopeptidase (LAPase) and β-glucosidase (BGase) differed, although higher EEA values were generally found during the productive seasons (Figures 3A–F). A sharp decrease in APase activity was registered during May 2015, coinciding with the above-mentioned low Chl *a* period (Figures 1B, 3A). Interestingly, APase-FL and LAPase-FL displayed a similar pattern with higher values during the productive period (Figures 3A,B; Supplementary Table S2). In contrast, LAPase per cell in both size-fractions increased in winter (Figure 3E) suggesting an increase in activity per cell associated with the use of complex organic matter during the non-productive period. BGase-FL increased through the end of summer while BGase-PA showed peaks during autumn and winter (Figure 3C) indicating a sequential utilization of carbohydrates by both size-fractions. Variability in APase activity was significantly correlated with changes in beta diversity of heterotrophic FL bacteria while changes in BGase and LApase activity were correlated with variability in beta diversity of heterotrophic PA bacteria (Mantel test; Supplementary Table S1). On the other hand, alpha diversity of both PA and FL bacteria was positively correlated with distinct EEAs (Supplementary Table S2). Our results suggest that changes in bacterial community structure in the PA and FL fractions are related to different exoenzymatic activities and therefore to different resource utilization by bacteria.

We tested how many model substrates (out of 31) were consumed by the bacterial community. The number of substrates used at each time point, indicative for functional metabolic potential and diversity of the community, varied over time (Figure 3G). It was decoupled in the two size-fractions studied, with a continuously increasing trend toward summer in the PA fraction and a later increase in the FL fraction (Figure 3G). Changes in the number of substrates used were positively correlated with alpha diversity of FL bacteria (Supplementary Table S2) and significantly correlated with differences in taxonomic composition in both size-fractions (Mantel tests; Supplementary Table S1). Thus, a close relationship between community composition and functional metabolic diversity of both PA and FL bacterial communities was observed. The number of substrates used was positively correlated to BA in both size-fractions and temperature and diversity in the PA fraction and negatively correlated with BP-FL per cell and BGE-FL (Figure 3G; Supplementary Table S2). These results point to a tendency for higher functional metabolic diversity associated with increases in abundance and taxonomic diversity that was slightly decoupled in both size-fractions. On one hand, PA bacteria displayed higher functional metabolic diversity during summer when phytoplankton blooms increase particle availability. On the other hand, FL bacteria would increase functional metabolic diversity when growing with low growth efficiency during periods of low resource availability in winter.

Among the substrates, polymer utilization relatively increased at the beginning of the phytoplankton blooms (early summer and spring in the PA and FL fractions, respectively) and was inversely correlated in both fractions to amino acid and carbohydrate utilization (higher during late summer and autumn and correlated with increased bacterial abundance and production; Figure 3H; Supplementary Table S2). Similarly, carboxylic acids and carbohydrate utilization occurred slightly earlier in the PA fraction than in the FL fraction (Figure 3H), indicating a probable sequential utilization of resources by PA and FL bacteria. It is important to note here that particle degradation may include, to some extent, a vertical component at the LMO site. Still, particles (mainly associated with surface phytoplankton blooms) are mostly present in surface waters from spring to autumn when thermal stratification (Legrand et al., 2015) increases the residence time of particles at the surface. Model polymer particles are typically degraded by a succession of bacteria with different functional properties, leading to a degrader, exploiter and scavenger behavior (Datta et al., 2016; Enke et al., 2019). This further results in hierarchical cross-feeding and sequential availability of substrates (Pontrelli et al., 2021). Our results highlight that such sequential resource utilization, at least in parts, shape the functional and potentially taxonomic diversity of microbial communities in natural environments both in PA and FL communities.

### Links Between Community Composition and Carbon Cycling at the Order Level

**Figure 4:**
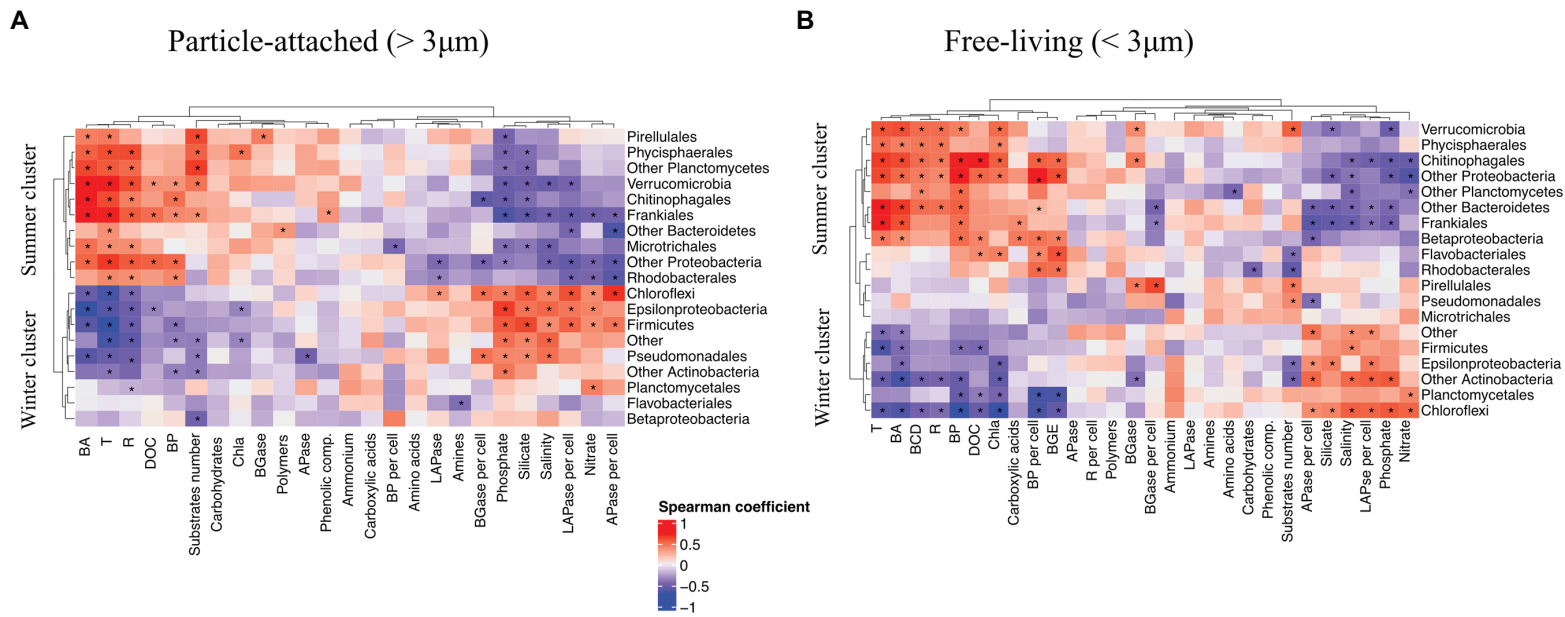
Spearman’s correlation between major bacterial groups and environmental variables, microbial abundance and activity in **(A)** PA fraction and **(B)** FL fraction. Asterisks denote significant correlations, *p* < 0.05.

To evaluate linkages between PA and FL bacterial taxa and carbon cycling across the year, we performed a correlation analysis between major bacterial groups and environmental and microbiological variables. This analysis showed that groups that maintained high relative abundance during the colder period of the year, from late autumn to beginning of spring, formed a “winter cluster” (including Firmicutes, Chloroflexi, Epsilonproteobacteria; Figure 4). The relative abundances of bacteria in this winter cluster was negatively correlated with Chl *a* and bacterial abundance and activity. Moreover, they tended to have positive correlations in both size-fractions with per cell EEAs [e.g., high LAPase_per cell and APase_per cell rates were correlated with increases in Epsilonproteobacteria, Firmicutes and Chloroflexi (Spearman’s rank correlation *p* < 0.05 as significance level)] and negative correlations with the number of substrates potentially used (Figure 4). In the absence of phytoplankton blooms during cold periods, the winter cluster bacteria would be adapted to low availability of labile organic matter resources along with high inorganic nutrient levels. Groups like Planctomycetes and Chloroflexi belonging to the winter cluster maintained high relative abundances during periods when low growth efficiencies were registered and less labile DOM (with a higher carbon to nitrogen ratio) is expected to be available throughout late phytoplankton bloom phase and the low productive periods (Goldman et al., 1992). Baltar et al. (2009) reported increased bacterial cell-specific hydrolytic enzyme activities with depth thought to reflect a reduced dissolved organic matter lability in deep compared to surface waters. In an analogous manner, we suggest that members of the winter cluster proliferate by taking advantage of complex polymeric organic matter, probably associated with the use of semi-labile to refractory organic matter remaining from the phytoplankton blooms earlier in the year.

In contrast, the “summer cluster” included bacterial groups that bloomed during the warmer period from late spring to beginning of autumn (e.g., Rhodobacterales, Chitinophagales, Verrucomicrobia and Bacteroidetes; Figure 4). The summer cluster bacteria likely benefit from the high resource availability during the spring and summer phytoplankton blooms and through their activity contribute to high secondary production and ectoenzymatic activities. Overall, in this system, Rhodobacterales, Flavobacteriales and Betaproteobacteria were associated with elevated growth efficiencies and specific production rates while elevated respiration rates and carbon demand coincided with elevated relative abundances of Verrucomicrobia and Phycisphaerales (Spearman test, *p* < 0.05; Figure 4). Members of the summer cluster were associated with the elevated values for utilization of carbohydrates derived from phytoplankton blooms (elevated BGase rates and carbohydrate utilization during spring and summer) that increased sequentially from the PA to the FL fraction, suggesting a resource partitioning between the two size-fractions. It is important to note here that this sequential utilization of resources may be related to some extent to the transfer of bacteria between size-fractions (e.g., detachment of PA bacteria or particle colonization by FL bacteria; Bižić-Ionescu et al., 2015, 2018).

Several summer cluster bacterial groups were correlated with the number of substrates used in each size-fraction (indicative for functional metabolic potential and diversity of the community; Spearman, *p* < 0.05, Figure 4) and we hypothesize that the elevated functional diversity measured during spring and summer reflects a more diverse set of metabolic strategies of summer cluster bacteria. These groups would have in common that they perform better under elevated temperature and high resource availability conditions and together can take advantage of the myriad of compounds released during and after phytoplankton blooms (Meon and Kirchman, 2001). Still, some interesting differences between the fractions were observed, such as FL and PA bacteria correlating with the utilization of carboxylic acids and carbohydrates, respectively (Figure 4). Even within the PA fraction there was a divergence in organic matter utilization between taxa, where, e.g., Frankiales correlated with the use of phenolic compounds, while Pirellulales correlated with carbohydrates (BGase activity).

Looking into the temporal pattern in bacterial substrate utilization, our results suggest that PA bacteria (e.g., Bacteroidetes) initially used complex polymers (Figures 3H, 4A), known to be released at the beginning of the blooms (Meon and Kirchman, 2001). The utilization of simpler compounds like carboxylic acids by FL bacteria appeared to occur later on (e.g., Frankiales and Betaproteobacteriales; Figures 3H, 4B). Such substrate-controlled succession is in accordance with the suggestion by Teeling et al. (2012) that, across phytoplankton blooms, the expression of distinct carbohydrate active enzymes and phosphate acquisition strategies promote the successive decomposition of algal-derived organic matter by Bacteroidetes and Proteobacteria. The summer cluster was also associated with elevated alkaline phosphatase activity (Figure 4), which is in line with the low phosphate concentrations in during summer (Figure 1A). During this period, competition for phosphorus between different microbial compartments is likely to increase, promoting the utilization of phosphorus from organic compounds (Thingstad et al., 1993; Moisander et al., 2003). In fact, the utilization of organically bound phosphorus by cyanobacteria has been suggested to favor filamentous cyanobacteria summer blooms in the Baltic Sea (Teikari et al., 2018). In this regard, the decrease in alkaline phosphatase activity during the previously mentioned “clear water-phase period” in May–June 2015 may be due to both the observed decrease in microplankton abundance and activity and a decrease in the competition for phosphorus.

Overall, our results showed that temporal dynamics in resource availability and environmental conditions promoted the development of two distinct clusters of bacteria in this temperate ecosystem. Yet, different taxa and different size-fractions within each cluster may contribute differently to organic matter utilization throughout the seasonal cycle. Interestingly, different ASVs of some orders (e.g., Pirellulales, Microtrichales) were grouped in a different cluster (i.e., “winter” or “summer” clusters) depending on the size-fraction they belonged to (Figure 4). This was corroborated by an analysis of specific ASVs belonging to these orders, showing that unique ASVs (not shared between size-fractions) were found in each size-fraction in these orders (Supplementary Figure S4). Thus, different bacteria belonging to the same order may occupy different ecological niches and thrive during different times of the year depending on the PA or FL life strategy displayed. This was the case of Pirellulales and Microtrichales: particle-attached ASVs in these orders were associated to the utilization of phytoplankton-derived organic matter but ASVs in the FL community nevertheless remained in the water column periods of low temperature and low production (Figure 4). Interestingly, Pirellulales were correlated with degradation of carbohydrates and high functional diversity independently of the life strategy displayed, which might be related to the maintenance of their functional capabilities in both size-fractions. On the other hand, FL Flavobacteriales and Betaproteobacteriales (from the summer cluster) were correlated with elevated growth efficiency and DOC concentration associated with phytoplankton-derived dissolved organic matter during spring and summer blooms while their PA counterparts relatively decreased in abundance during the summer bloom, probably due to the increase in Chitinophagales better performing as opportunistic bacteria taking advantage of particles (Figure 4).

### Linking Bacterial Populations (Amplicon Sequence Variants) and Community Function

In general, ASVs of heterotrophic bacteria present in both the FL and PA fractions had higher relative abundance than those unique for each of the size-fractions (Supplementary Figure S3C). A few exceptions to this pattern were found (e.g., spring 2015 and 2016) when an increase in the relative abundance of unique ASVs coincided with increases in alpha diversity in that respective size-fraction (Supplementary Figure S3B). To obtain further insight into the potential linkages between the most abundant PA and FL bacteria and biological and environmental variables across the year, we performed a detailed analysis of the spatiotemporal distribution of the dominant ASVs in the two size-fractions.

**Figure 5:**
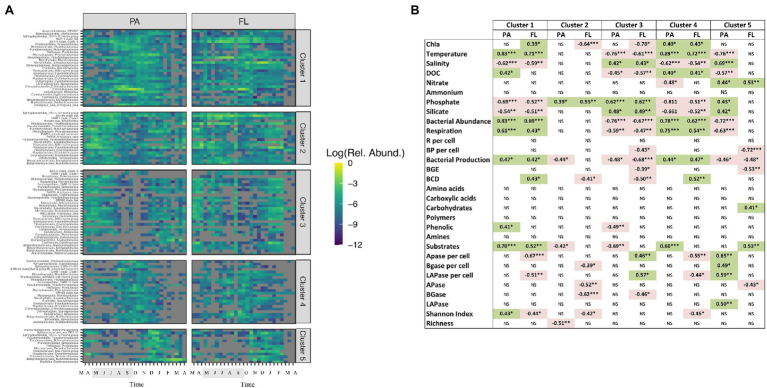
**(A)** Temporal evolution of the 100 most abundant bacterial ASVs in the PA and FL size-fractions in clusters defined by Euclidean distances. The shaded period in the x-axis corresponds to summer. **(B)** Spearman’s correlations between the abundance of the different clusters generated in panel **A** and environmental variables and microbial abundance and activity in the two size-fractions studied. NS: not significant. Green background: positive correlation, red background: negative correlation. *p < 0.05; **p < 0.01; and ***p < 0.001.

The 100 most abundant ASVs in each of the PA and FL fractions (a total of 152 ASVs) grouped into five different clusters attending to their temporal dynamics in relative abundance (Figure 5A). ASVs in the clusters 1 and 4 increased their relative abundance from virtually absent to elevated abundances during the summer and autumn periods (most clearly in the PA fraction). This suggests that the increase in bacterial abundance observed during summer and autumn may at least be partially related to the increase in relative abundance of ASVs from the rare biosphere (for LMO, previously described as taxa with a relative abundance ≤0.01% of total community, Lindh et al., 2015). In accordance with our results, Lindh et al. (2015) showed that most dominant bacterial populations (then denoted as operational taxonomic units, OTUs, based on clustering of 16S rRNA gene amplicon sequences based on 97% sequence identity) in this system do not stay abundant, but actually become part of the seed bank of rare taxa for extended time periods, suggesting that substantial portions of the bacterial community are sensitive to environmental changes or disturbances. The precise whereabouts of individual bacterial populations when not detected by 16S rRNA gene amplicon sequencing remains intriguingly unknown; we think it is likely that some populations (potentially a majority?) remain as rare components of the pelagic plankton, whereas others may be seeded in from deeper waters during spring or autumn overturn, and others again may be introduced from more distant surface water locations. In this context, it is important to recognize that the Baltic Sea microbiome does not represent a mix of freshwater taxa brought in by rivers along with taxa coming in with seawater through the Danish straits, but has evolved to be composed of taxa matching the brackish conditions (Hugerth et al., 2015).

Most of the 152 ASVs were observed in both the FL and the PA fractions (Figure 5A), in accordance with previous studies showing that FL and PA bacterial communities to a large extent contain similar populations (Salazar et al., 2016). Interestingly, in our data set, the ASVs followed slightly different temporal dynamics in each fraction (Figure 5A) and in general the abundance of a specific ASV in the FL fraction showed low correlation with that of the same ASV in the PA fraction (Supplementary Figure S5). In fact, the 152 ASVs displayed a sequential transition from one life strategy to the other, possibly due to the colonization of new particles coming from seasonal phytoplankton blooms (Pedrós-Alió and Brock, 1983; Ghiglione et al., 2007; Crespo et al., 2013; Bižić-Ionescu et al., 2018) or to detachment from particles (Bižić-Ionescu et al., 2015). As an example, ASVs in cluster 1 were more abundant first in the PA fraction (during summer and autumn) and later on (late summer, autumn and winter) in the FL fraction (Figure 5A). Interestingly, different clusters were associated to different community functions (Figure 5B). While some of the most abundant PA ASVs in the Baltic Sea thrived coinciding with elevated cell-specific ectoenzymatic activity in winter (e.g., several Pseudomonadales and Betaproteobacteriales from cluster 5), other FL ASVs thrived in summer when functional diversity increased (i.e., several Frankiales and Microtrichales from cluster 1; Figure 5B).

## Conclusion

We have presented here the first work jointly determining the dynamics in abundance, activity (production, respiration, substrate utilization and exoenzymatic activity) and taxonomy of bacteria in the FL and PA fractions over a full yearly cycle. Overall, our analysis across seasons allowed us to postulate that the temporal dynamics of bacteria with distinct life strategies are remarkably different. This probably depended on temporal changes in their corresponding resource supply (i.e., availability of different particles and algal cells versus DOM) and the differential control exerted by environmental factors (e.g., temperature) on bacteria in the FL and PA fractions. We have uncovered both a probable sequential utilization of resources and a pronounced substrate partitioning, not only between bacteria with different life strategies (PA versus FL) but also between members of different bacterial orders detected at the ASV level. Collectively, our analysis revealed how the intricate links between taxonomy, abundance and activity, and carbon budgets dynamically change over a yearly cycle among PA and FL bacteria in a temperate ecosystem. Our work suggests that future studies in marine microbial ecology aiming at understanding the role of microbes in biogeochemical cycling in general and the biological carbon pump in particular should take into account the lifestyles of different taxa and/or dominant populations and their contribution to the degradation of the complete spectrum of particulate and dissolved organic matter in a continuously changing environment.

## Data Availability Statement

The data presented in the study are deposited in the EMBL-EBI European Nucleotide Archive repository (doi: https://www.ebi.ac.uk/ena), accession number PRJEB52496.

## Author Contributions

SM-G and JP conceived the research plan and wrote the paper. All authors performed the research, analyzed the data, provided critical comments and insights on the manuscript, and contributed to editing the paper. All authors contributed to the article and approved the submitted version.

## Funding

This research was supported by Grant PID2019-110011RB-C33 funded by MCIN/AEI/10.13039/501100011033 and “ERDF A way of making Europe.” The research was supported by the Swedish Research Council FORMAS Strong Research environment EcoChange (Ecosystem dynamics in the Baltic Sea in a changing climate) to CL and JP and by the Linnaeus University Center for Ecology and Evolution in Microbial model Systems (EEMiS). SM-G was supported by a Marie Curie-IEF fellowship from the European Research Council. CB was supported by the Ministry for Science and Culture of Lower Saxony Vorab grant “Ecology of Molecules, EcoMol”.

## Conflict of Interest

The authors declare that the research was conducted in the absence of any commercial or financial relationships that could be construed as a potential conflict of interest.

## Publisher’s Note

All claims expressed in this article are solely those of the authors and do not necessarily represent those of their affiliated organizations, or those of the publisher, the editors and the reviewers. Any product that may be evaluated in this article, or claim that may be made by its manufacturer, is not guaranteed or endorsed by the publisher.
