# Supplementary material for: Seasonal Dynamics in Carbon Cycling of Marine Bacterioplankton Are Lifestyle Dependent
Source: Front Microbiol. 2022 Jul 5;13:834675. doi: 10.3389/fmicb.2022.834675 (PMC9533715; doi:10.3389/fmicb.2022.834675)
Supplement: Supplementary file 1 [file Table_1.pdf]

Table S1. Mantel tests coefficients between the change in bacterial community composition and the change in environmental variables, and bacterial abundance and activity in the two size-fractions studied: > 3µm (PA) and < 3µm (FL), computed from Mantel test., n=27. NS: Not significant; “NS”: p<0.05; *: p<0.05, **: p<0.01

	DATASET	
**VARIABLE**	FL	PA
Chl a	NS	NS
T	NS	**0.24****
Salinity	NS	**0.21***
DOC	NS	NS
NO3	**0.33****	**0.23***
NH4	NS	NS
PO4	NS	**0.36****
SiO2	NS	**0.27***
BA	NS	NS
R	NS	NS
R_per cell	**0.32****	-
BP	NS	NS
BP_per cell	NS	NS
BGE	NS	-
BCD	NS	-
Amino acids	NS	NS
Carboxylic acids	NS	NS
Carbohydrates	NS	NS
Polymers	NS	NS
Phenolic compounds	**0.26***	NS
Amines	NS	**0.38****
Substrates	**0.15***	**0.20***
Apase activity	NS	NS
Apase activity per cell	**0.41***	NS
Bgase activity	NS	NS
Bgase activityper cell	NS	**0.39***
LAPase activity	NS	NS
LAPase activity per cell	NS	**0.46***
