# Supplementary figures and images for: Seasonal Dynamics in Carbon Cycling of Marine Bacterioplankton Are Lifestyle Dependent
Source: Front Microbiol. 2022 Jul 5;13:834675. doi: 10.3389/fmicb.2022.834675 (PMC9533715; doi:10.3389/fmicb.2022.834675)

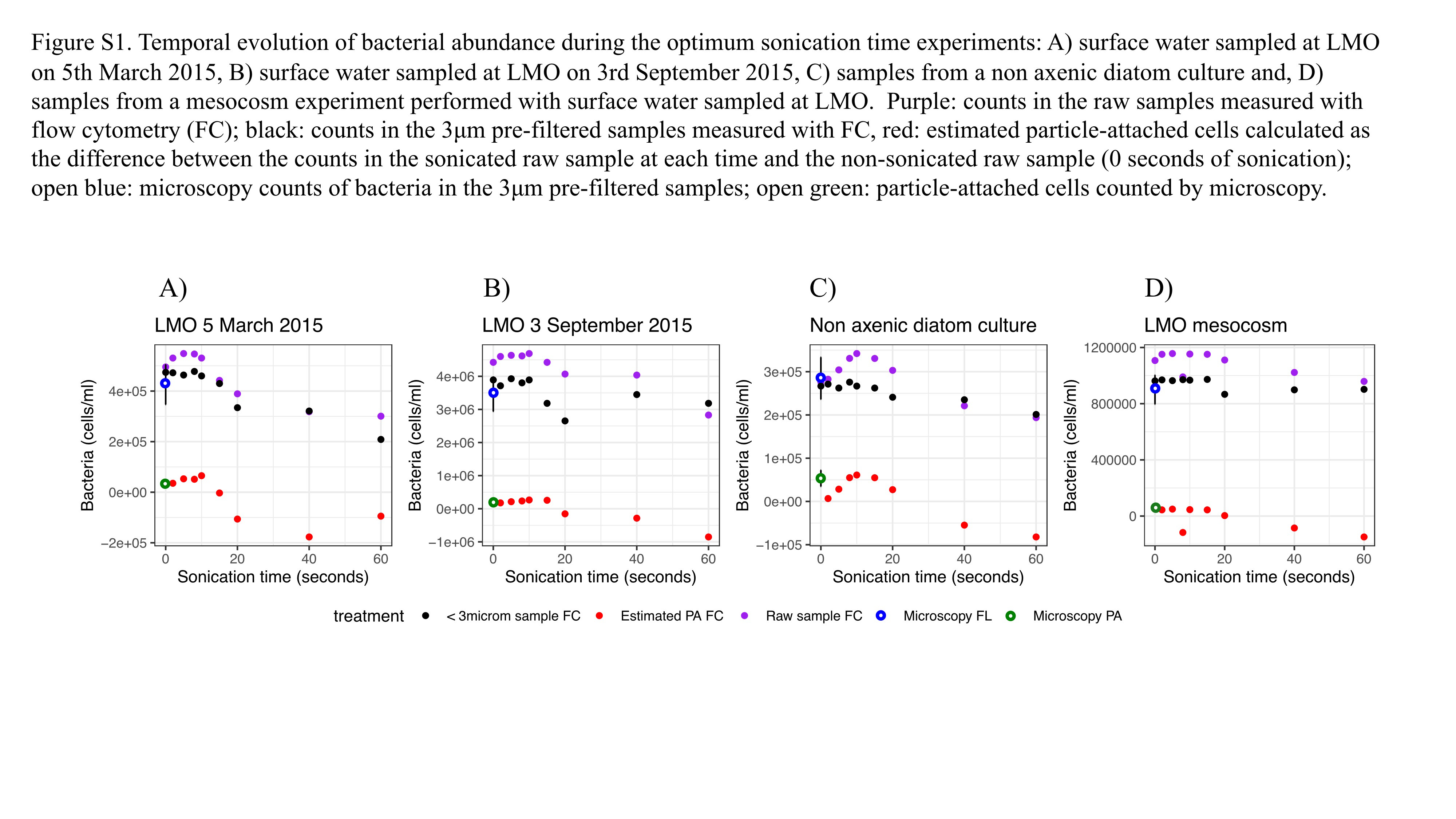

Supplement: Supplementary file 3 [file Image_1.TIF]

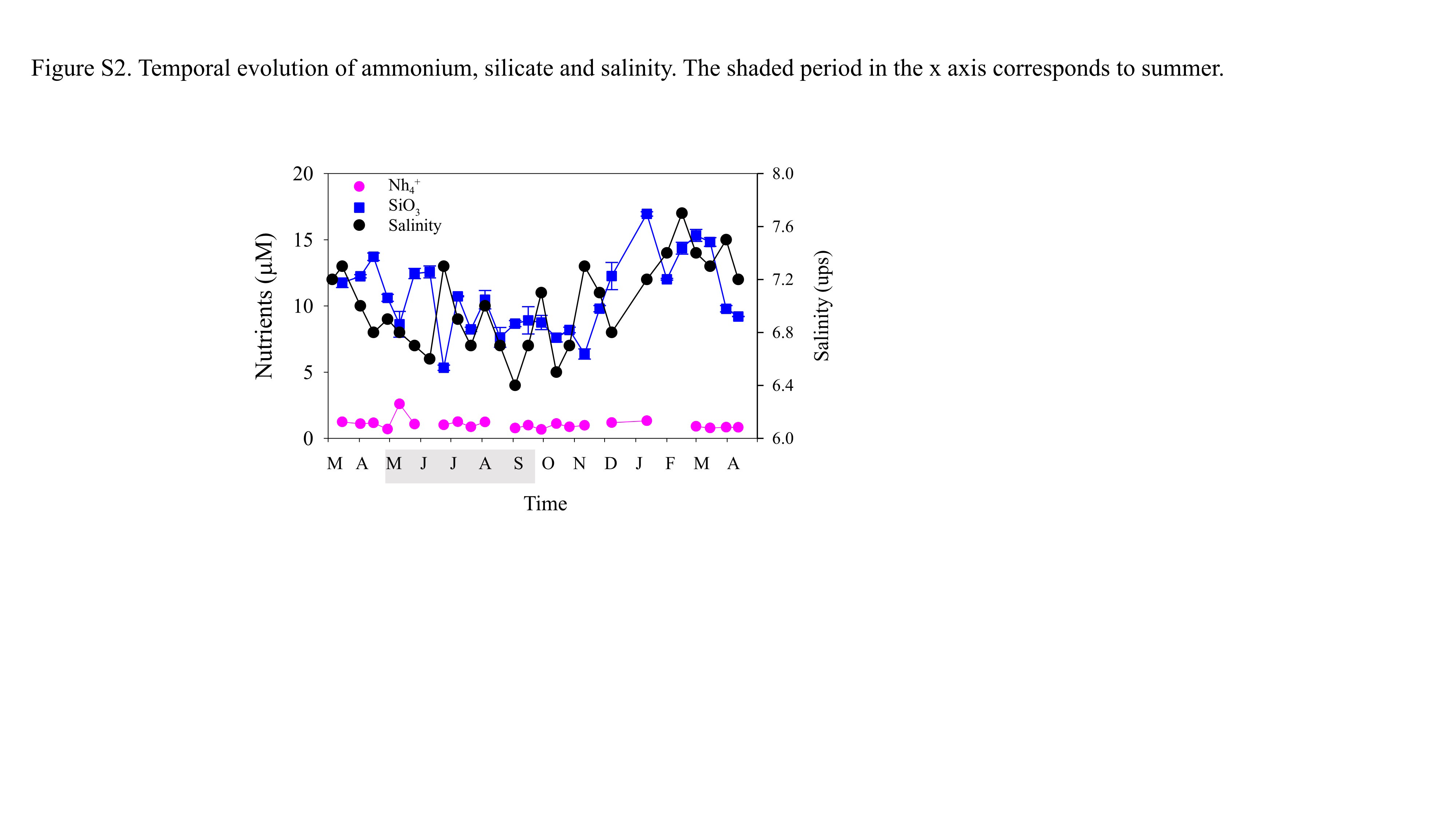

Supplement: Supplementary file 4 [file Image_2.TIF]

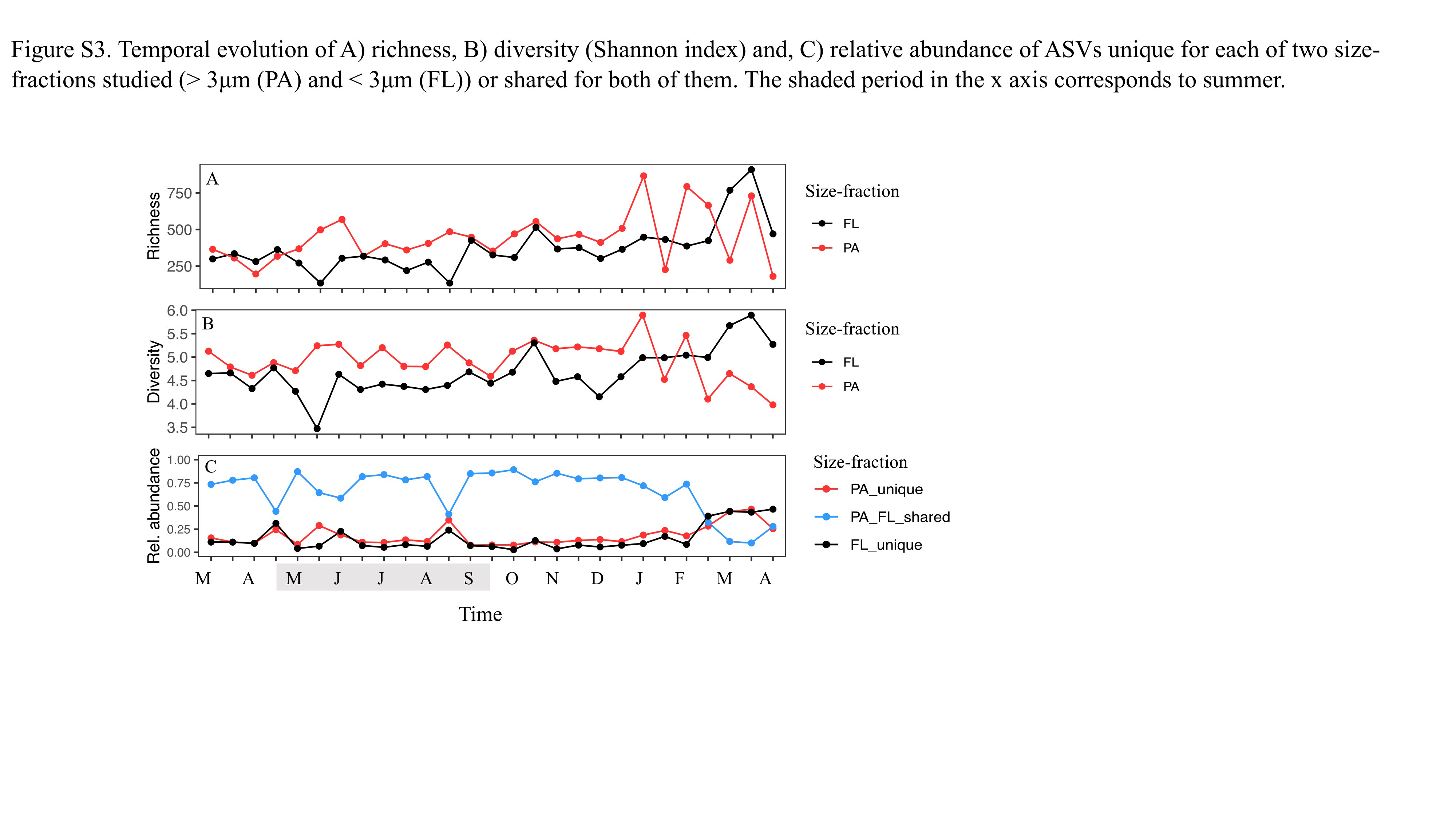

Supplement: Supplementary file 5 [file Image_3.TIF]

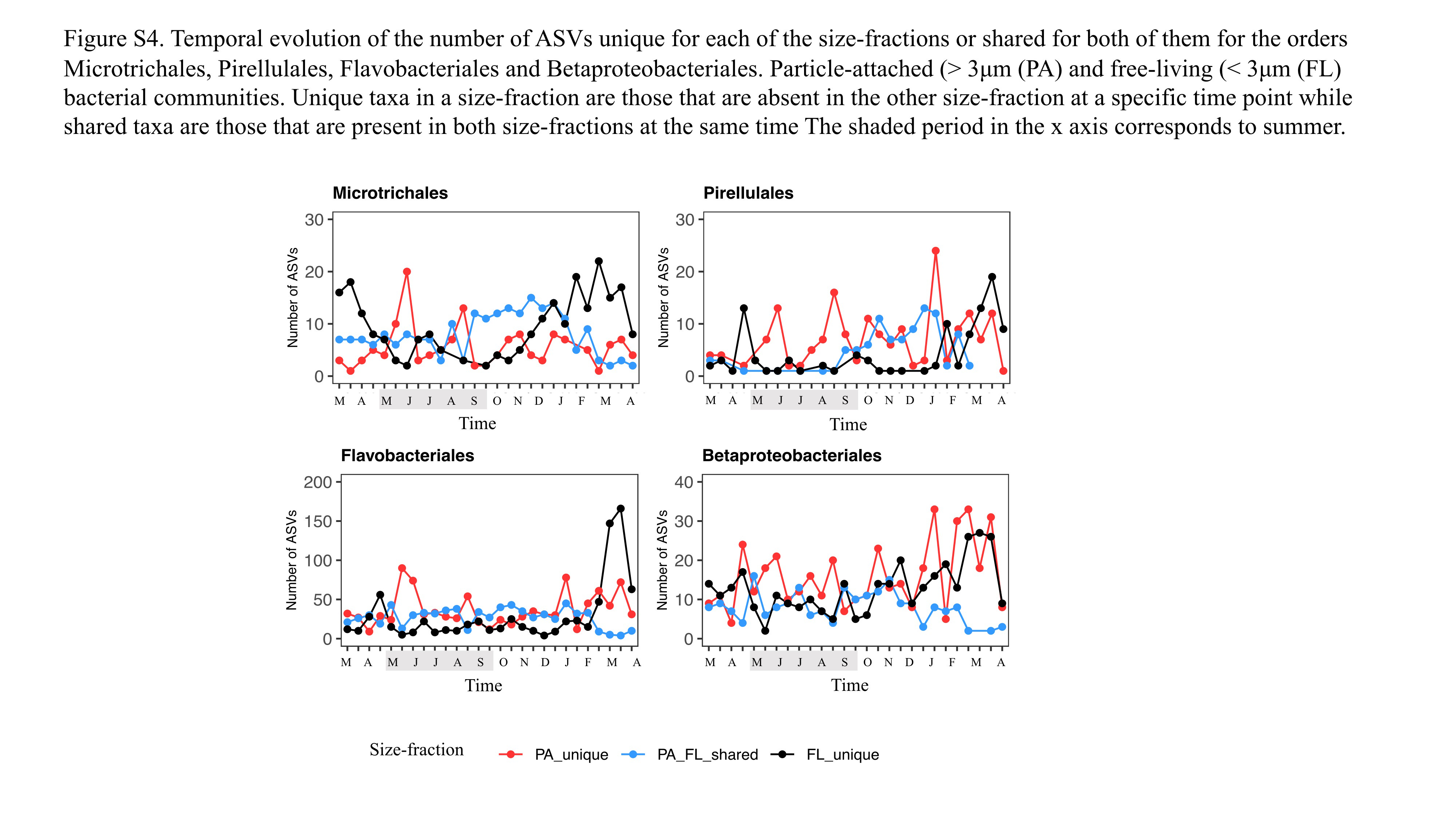

Supplement: Supplementary file 6 [file Image_4.TIF]

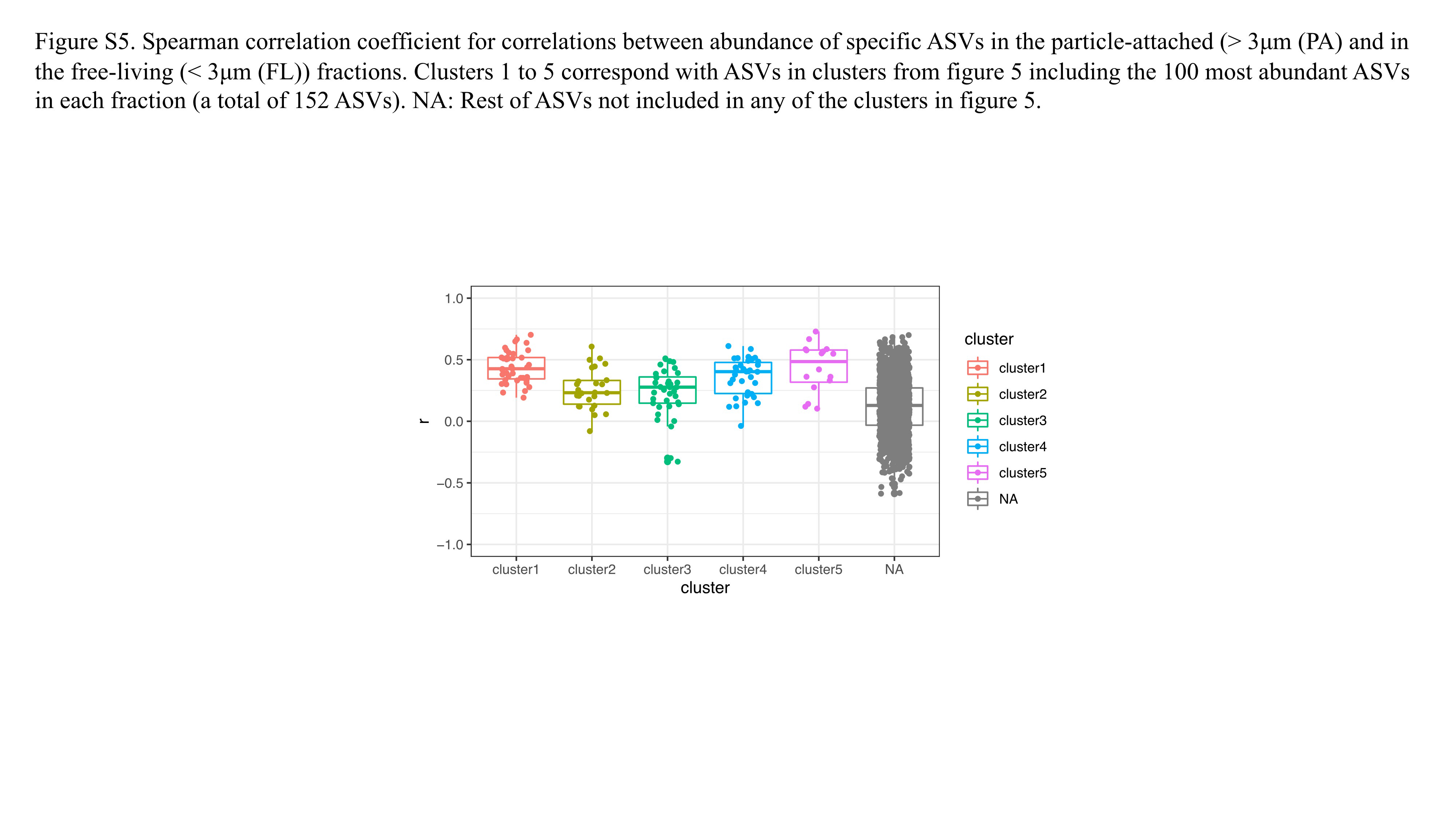

Supplement: Supplementary file 7 [file Image_5.TIF]
