# Supplementary material for: Seasonal Dynamics in Carbon Cycling of Marine Bacterioplankton Are Lifestyle Dependent
Source: Front Microbiol. 2022 Jul 5;13:834675. doi: 10.3389/fmicb.2022.834675 (PMC9533715; doi:10.3389/fmicb.2022.834675)
Supplement: Supplementary file 2 [file Table_2.pdf]

Table S2. Spearman correlation coefficient for correlations between environmental variables and bacterial abundance, activity, substrate use and extracellular enzyatic activity (EEA) for particle-attached (> 3µm (PA) and free-living (< 3µm (FL) bacterial communities. NS: not significant. Green background: positive correlation, red background: negative correlation. * p<0.05; ** p<0.01; *** p<0.001

		Environmental variables								Bacterial abundance and activity								Substrate use							EEA						Tanonomical composition
		Chla	T	Sal	DOC	NO3	NH4	PO4	SiO2	BA	Synech.	R	R per cell	BP	BP per cell	BGE	BCD	Amino acids	Carbox. acids	Carbohydrates	Polymers	Phenolic	Amines	Susbstrates number	APase	Apase per cell	BGase	Bgase per cell	LAPase	LAPase per cell	Richness
Environmental variables	Chla																														
	T	0.39*																													
	Sal	NS	-0.71***																												
	DOC	NS	0.60**	-0.52**																											
	NO3	NS	-0.51**	NS	NS																										
	NH4	NS	NS	NS	NS	NS																									
	PO4	-0.51**	-0.81***	0.53**	NS	0.68***	NS																								
	SiO2		-0.70***	NS	NS	0.66***	NS	0.82***																							
PA	BA	NS	0.87***	-0.62**	0.61**	NS	NS	-0.70**	-0.57*																						
	Synech.	NS	NS	NS	NS	NS	NS	NS	NS	NS																					
	R	0.69***	0.84***	-0.61***	0.68***	-0.53**	NS	-0.76***	-0.60**	0.69**	NS																				
	BP	NS	0.58**	NS	0.50**	-0.48*	NS	-0.50*	NS	0.59**	NS	0.58**																			
	BP per cell	NS	NS	NS	NS	NS	NS	NS	NS	NS	0.6**	NS		0.64**																	
	Amino acids	NS	NS	NS	NS	0.58**	NS	0.47*	0.52**	NS	0.47*	NS		NS	NS																
	Carbox. acids	-0.42*	NS	NS	NS	NS	NS	NS	NS	NS	NS	NS		NS	NS			NS													
	Carbohydrates	NS	0.42*	-0.39*	NS	NS	NS	NS	NS	0.60**	NS	NS		0.49*	NS			NS	-0.48*												
	Polymers	NS	NS	NS	NS	NS	NS	NS	NS	NS	NS	NS		NS	NS			-0.67***	NS	-0.43*											
	Phenolic	NS	NS	NS	NS	NS	NS	NS	NS	NS	NS	NS		NS	NS			NS	NS	NS	NS										
	Amines	NS	NS	NS	NS	NS	NS	NS	NS	NS	NS	NS		NS	NS			NS	NS	NS	NS	NS									
	Substrates number	NS	0.50*	NS	NS	NS	NS	NS	NS	0.64**	NS	0.40*		NS	NS			NS	NS	NS	NS	0.56**	0.42*								
	APase	NS	NS	NS	NS	NS	NS	NS	NS	NS	NS	NS		NS	NS			NS	NS	NS	NS	NS	NS	NS							
	Apase per cell	NS	-0.55*	0.48*	-0.65***	NS	NS	NS	0.46*	-0.48***	NS	NS		NS	NS			NS	NS	-0.47***					0.68**						
	BGase	NS	NS	NS	NS	NS	NS	NS	NS	NS	NS	NS		NS	NS			NS	NS	NS	NS	NS	NS	0.38*	NS	NS					
	Bgase per cell	NS	NS	NS	NS	NS	NS	NS	NS	NS	NS	NS		NS	NS			NS	NS	NS	NS	NS	NS	NS	NS	NS	0.58**				
	LAPase	NS	NS	NS	NS	NS	NS	NS	NS	NS	NS	NS		NS	NS			NS	NS	NS	NS	NS	NS	NS	NS	NS	NS	0.44*			
	LAPase per cell	NS	-0.55*	NS	NS	NS	NS	NS	NS	-0.59**	NS	NS		NS	NS			NS	NS	NS	NS	NS	NS	NS	NS	0.49*	NS	0.53*	0.91***		
	Richness	NS	NS	NS	NS	NS	NS	NS	NS	NS	NS	NS		NS	NS			NS	NS	NS	NS	NS	0.43*	NS	NS	NS	0.49*	0.53*	NS	NS	
	Diversity (Shannon)	NS	NS	NS	NS	NS	0.46*	NS	NS	NS	NS	NS		NS	NS			NS	NS	NS	NS	NS	NS	0.49*	NS	NS	0.50**	NS	NS	NS	0.58**
FL	BA	0.52**	0.89***	-0.58	0.47*	-0.55**	NS	-0.86***	-0.74***																						
	Synech.	0.43*	0.77***	-0.55	0.50**	NS	NS	-0.61**	-0.56**	0.79***																					
	R	0.38*	0.79***	-0.42	NS	-0.49*	NS	-0.81***	-0.70***	0.84***	0.55**																				
	R per cell	NS	0.36*	NS	NS	-0.48	NS	-0.57**	-0.50**	0.38*	NS	0.76***																			
	BP	0.57**	0.80***	-0.55	0.56**	-0.78***	NS	-0.83***	-0.67***	0.77***	0.49**	0.71***	0.42*																		
	BP per cell	0.44*	NS	NS	0.37*	-0.70**	NS	-0.43*	NS	NS	NS	NS	NS	0.74***																	
	BGE	0.41*	NS	NS	0.44*	-0.41*	NS	NS	NS	NS	NS	NS	NS	0.46*	0.74***																
	BCD	0.40*	0.80***	-0.44	NS	-0.54**	NS	-0.83***	-0.69***	0.85***	0.56**	0.99***	0.76***	0.76***	NS	NS															
	Amino acids	NS	NS	NS	NS	NS	NS	NS	NS	NS	NS	NS	NS	NS	NS	NS	NS														
	Carbox. acids	NS	NS	NS	NS	NS	NS	NS	NS	NS	NS	NS	NS	NS	NS	NS	NS	NS													
	Carbohydrates	NS	NS	-0.36*	NS	NS	NS	NS	NS	NS	NS	NS	NS	NS	NS	NS	NS	NS	NS												
	Polymers	NS	NS	NS	NS	NS	NS	NS	NS	NS	NS	NS	NS	NS	NS	NS	NS	-0.46*	NS	-0.61***											
	Phenolic	NS	NS	NS	NS	NS	NS	NS	NS	NS	NS	NS	NS	NS	NS	NS	NS	NS	NS	NS	NS										
	Amines	NS	NS	NS	NS	NS	NS	NS	NS	NS	NS	NS	NS	NS	NS	NS	NS	NS	NS	NS	NS	NS									
	Substrates number	NS	NS	NS	NS	NS	NS	NS	NS	0.41*	0.46*	NS	NS	NS	-0.44*	-0.39*	NS	NS	NS	NS	-0.40*	NS	0.56**								
	APase	0.40*	NS	NS	NS	NS	NS	NS	NS	NS	NS	NS	0.55**	NS	NS	NS	0.39*	-0.45*	NS	NS	NS	NS	NS	NS							
	Apase per cell		-0.62***	0.56**	-0.46*	NS	NS	0.40*	0.42*	-0.64***	-0.63***	-0.38*	NS	-0.36*	NS	NS	-0.37*	NS	NS	NS	NS	NS	NS	NS	0.66***						
	BGase	0.47*	NS	NS	0.40*	NS	-0.46*	-0.54**	NS	0.50**	0.40*	0.43*	NS	0.39*	NS	NS	0.45*	NS	NS	NS	NS	NS	NS	NS	0.40*	NS					
	Bgase per cell	NS	NS	0.39*	NS	NS	NS	NS	NS	NS	NS	NS	NS	NS	NS	NS	NS	NS	NS	NS	NS	NS	NS	NS	NS	NS	0.74***				
	LAPase	NS	NS	NS	NS	NS	0.45*	NS	NS	NS	NS	0.38*	NS	NS	NS	NS	0.37*	NS	NS	NS	NS	NS	0.34*	NS	NS	NS	NS	NS			
	LAPase per cell	-0.42*	-0.52***	0.53*	0.40*	NS	0.53*	0.40*	NS	-0.53**	-0.76***	NS	NS	-0.52**	NS	NS	-0.34*	NS	NS	NS	NS	NS	NS	NS	NS	0.53**	-0.35*	NS	0.52**		
	Richness	NS	-0.44*	0.46*	NS	NS	NS	NS	NS	NS	NS	NS	NS	NS	NS	NS	NS	NS	NS	NS	NS	NS	NS	NS	NS	NS	NS	0.60***	NS	NS	
	Diversity (Shannon)	NS	-0.46*	0.54**	NS	NS	NS	NS	NS	NS	NS	NS	NS	NS	NS	NS	NS	NS	NS	NS	NS	NS	NS	NS	NS	0.39**	NS	0.65***	NS	NS	0.80***
